# Fabrication of a state of the art mesh lock polymer for water based solid free drilling fluid

**DOI:** 10.1038/s41598-021-98379-w

**Published:** 2021-09-22

**Authors:** Chaoqun Wang, Wei Ding

**Affiliations:** 1 College of Chemistry and Chemical Engineering, Northeast Petroleum University, Daqing, 163318 Heilongjiang China; 2 Department of Oil Field Chemistry, China Oilfield Services Ltd., Tianjin, 300450 China

**Keywords:** Polymer chemistry, Chemical synthesis, Chemistry, Energy

## Abstract

Polymers are used widely in various kinds of drilling fluid to maintain the proper rheological properties. However, most of them are not available for high-temperature or salt solutions due to poor temperature and salt resistance. To ameliorate the temperature and salt resistance of polymer used in the solid-free water-based drilling fluid, a novel polymer with a kind of "Mesh-Lock" reinforced network cross structure, named PLY-F [main monomer acrylic acid (AA), acrylamide (AM), functional monomers 2-acrylamide-2-methylpropanesulfonic acid (AMPS) *N*-vinylpyrrolidone (NVP) and C_16_DMAAC] were prepared through free radical polymerization of an aqueous solution of organic cross-linking agent pentaerythritol triallyl ether (PTE) as a cross-linking system, Potassium persulfate (KPS) and sodium bisulfite as the initiator for the first time. The surface morphology, crosslinking architecture and temperature and salt resistance of the PLY-F were fully characterized with several means including SEM, FT-IR, ^13^CNMR, dynamic rheology, and long-term thermal stability. The SEM observation indicated that the PLY-F exhibits a regular “Mesh-Lock” reinforced network cross structure. FT-IR, ^13^CNMR analysis indicated that the characteristic functional groups of each monomer such as AM, AA, AMPS and NVP were all together in the polymer. The results show that the apparent viscosity retention rate of the PLY-F in the potassium formate solution (with a density of 1.3 g/cm^3^) was more than 80% after heat rolling for 72 h at 200 °C and the plastic viscosity retention rate reached 90.3%. Moreover, the salt resistance of the polymer can reach the density of 1.4 g/cm^3^ (potassium formate solution) under 200 °C and the temperature resistance can reach 220 °C under the density of 1.3 g/cm^3^ (potassium formate solution). Besides, the PLY-F still has good rheological properties in other saturated solutions (NaCl, HCOONa) under 210 °C.

## Introduction

At present, the temperature and salt-resistant polymers applied in the oilfield drilling process are mainly focused on two aspects: functional polymers with EOR (Enhanced Oil Recovery) technology and polymers for drilling fluid technology^1–3^.

In order to maintain the stability of the polymer used in oilfield recovery, many function polymers have been studied. These functional groups include biomonomers, metal cross-linking and hydrophobic monomers^4–6^. Similarly, polymers are used as key treatment agents which can improve the viscosity and filtration reduction at HTHP (High Temperature and High Pressure) condition of drilling fluid system to adjust the rheological properties of water-based drilling fluid^7–12^.

The methods as mentioned in the EOR technology can improve the tolerance of polymer to temperature and salinity under reservoir conditions. However, it can hardly work in the application environment of the drilling fluid system.

On one hand, the salinity in the drilling fluid system is higher. For considering the requirement of density, the drilling fluid system usually needs to add 40–50% of all kinds of aggravated salt, which is much larger than the salinity in reservoir conditions. On the other hand, the drilling fluid system faces higher temperatures, especially for high-temperature deep wells (T > 200 °C), polymers are difficult to stabilize under harsh conditions.

In terms of the polymer-modified in drilling fluid conditions, AMPS/NVP/AA are usually chosen to copolymerize with AM^13,14^ and further, a growing number of functional monomers have been selected, such as IA (itaconic acid)/SSS (Sodium p-styrenesulfonate)/APEG (alllyl alcohol polyoxyethylene ether)/AHPS (3-allyl oxy-2-hydroxy-1-propanesulfonic acid) are used to improve the salt-resistant properties of polymers^15,16^.

Some other synthetic polymers which have good performance under high temperature and salinity have been reported: a kind of amphoteric polyelectrolyte that can be used in KCl-saturated drilling fluid was prepared using AM/AMPS/TAC^17^. A filtrate reducer was made with the monomers of AM/AMCnS/AOIAS. It can reduce fluid loss and control viscosity significantly at high temperatures up to 220 °C and salinity tolerance to saturation of NaCl^18^. A tetrapolymer of AM/APMS/NVP/AA was used as the filtrate reducer in solid-free water-based drilling fluid of Iran reservoirs at 150 °C^19^. Besides that, a novel hydrophobic associated polymer-based nano-silica composite with core–shell structure was prepared with AM/AMPS/MA/St and nano-silica via inverse micro-emulsion polymerization^20,21^.

Most of the above studies are aimed at the work of temperature and salt resistant polymer with the function of filtration reducer. Some discussed the addition of clay to the system, and some consider the stability of polymers in the presence of high temperature or high salt conditions alone. However, there are few studies of polymers used as the viscosifier at high temperature and high salt, especially on water-based drilling fluid without any clay^22,23^.

**Figure 1:**
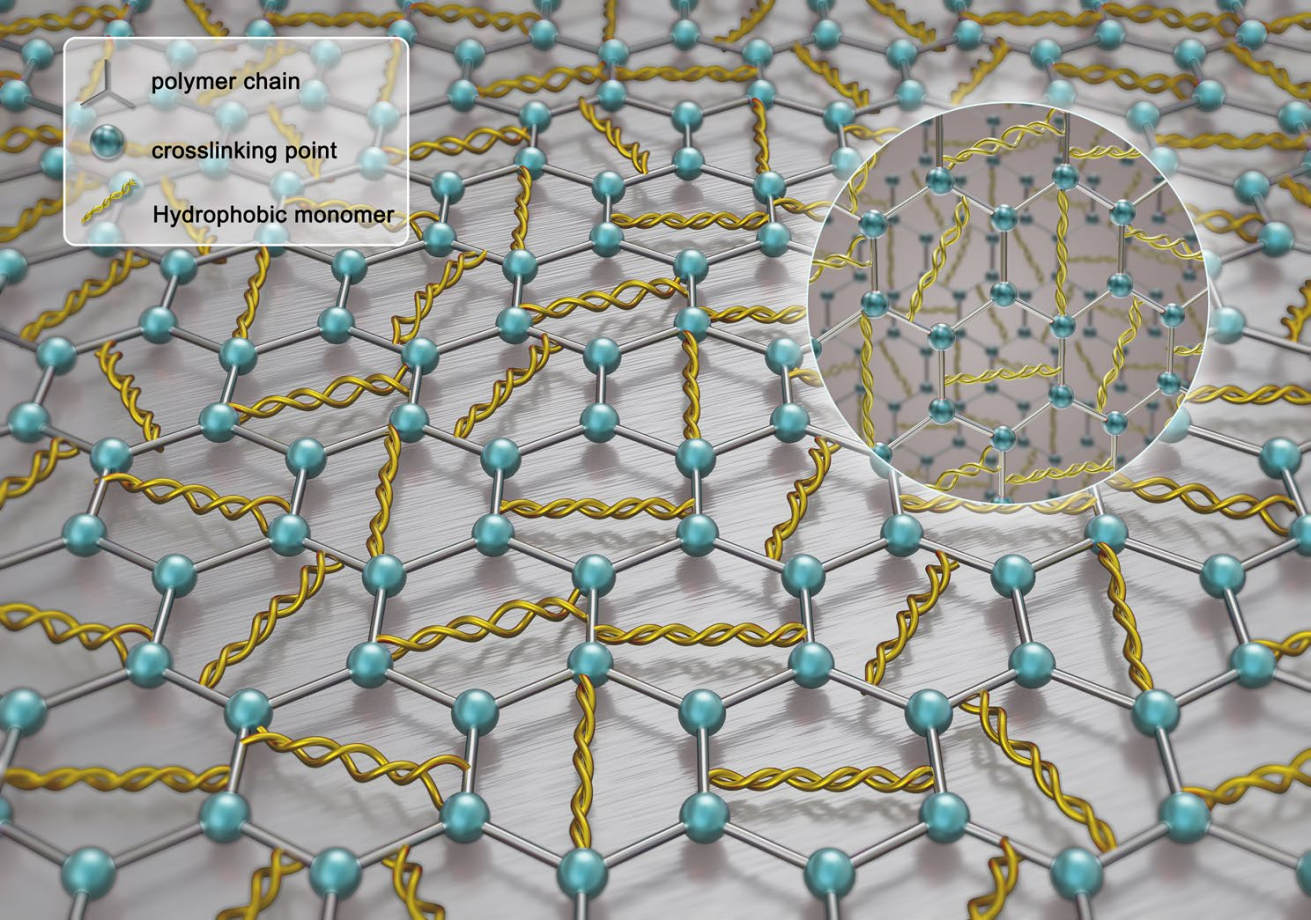
"Mesh-Lock" simulation diagram of PLY-F.

In this study, a kind of "Mesh-Lock" reinforced network cross polymer (as shown in Fig. 1) named PLY-F which can be used as the viscosifying agent in the solid-free water-based drilling fluid was researched. It was constructed by means of free-radical polymerization of acrylamide, 2-acrylamide-2 methacrylic acid, acrylic acid, *N*-vinyl pyrrolidone, pentaerythritol allyl ether and cetyl dimethylallyl ammonium chloride.

## Materials and methods

### Materials

Acrylamide (AM, 99%), acrylic(AA, 98%), *N*-vinyl pyrrolidone (NVP, 98%), 2-acrylamide-2-methylpropanesulfonic acid (AMPS, industrial grade), pentaerythritol triallyl ether (PTE, 99%), cetyl dimethylallyl ammonium chloride (C_16_DMAAC, 80%), potassium formate (HCOOK, industrial grade), sodium hydroxide (NaOH, AR), sodium carbonate (Na_2_CO_3_, AR), potassium persulfate (KPS, AR), sodium bisulfite (NaHSO_3_, AR), all the above materials were purchased from Shanghai Macklin Biochemical Co. LTD, China and were used without any further purification. Seawater is prepared by artificial seawater as Table 1.

**Table 1:** Artificial seawater formulation.

Substances	Concentration (g/L)
NaCl	21.86 ± 0.01
Na_2_SO_4_	3.23 ± 0.01
MgCl_2_	4.53 ± 0.01
CaCl_2_	0.93 ± 0.01
KCl	0.64 ± 0.01
NaHCO_3_	0.17 ± 0.01
Na_2_CO_3_	0.02 ± 0.01

### Synthesis of polymer

**Figure 2:**
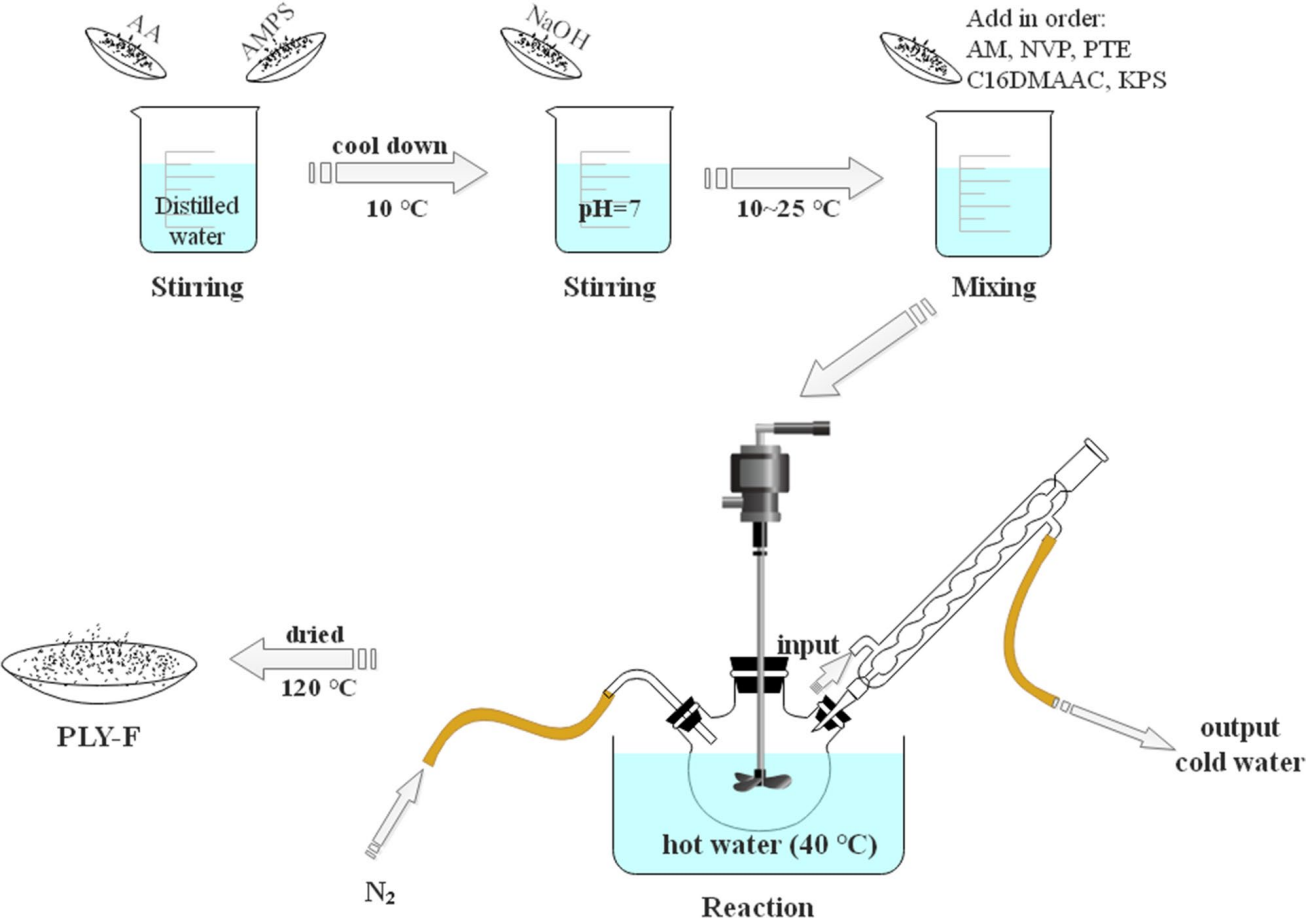
The prepare methodology flow of PLY-F of PLY-F.

Firstly, a certain amount of monomer AA (8.5 g), AMPS (1.8 g)was dissolved in 80 g of distilled water. After that, the solution was cooled to 10 °C, and then 4.8 g NaOH was added slowly when the solution temperature was kept at not more than 25 °C during this period. Other comonomers (4.2 g AM, 2.74 g NVP, 0.02 g PTE, 0.3 g C_16_DMAAC) and initiator 0.005 g KPS were added to the solution in turn. Pour the solution to a clean and dry detachable three-necked flask and heated to 40 °C. In the meantime, the solution began to react after adding 0.003 g NaHSO_3_ with nitrogen for 30 min. Secondly, release circulating water from the jacket and keep the reaction under adiabatic condition for 7 h to obtain colloidal product and it was granulated under 120 °C, dried for 5 h and crushed to white powder polymer product (named as PLY-F). The prepared methodology flow of PLY-F is as shown in Fig. 2^24–26^.

### Characterization

A Nicolet iS5 spectrometer manufactured by Thermo fisher was used to measure the Fourier transformation infrared (FT-IR) spectra of the PLY-F.

^13^CNMR spectroscopy was performed on a Bruker-AV300 ^13^CNMR system operating at 9.4 T with corresponding ^13^C resonance frequencies of 100.6 MHz using a 5 mm one NMR™ probe under 25 °C.

The SEM of PLY-F was tested by SU8010 which was manufactured by the Hitachi Company of Japan. firstly, the polymer was prepared into a solution of 1% mass concentration, frozen with liquid nitrogen for 48 h, and then vacuum dried to obtain the sample. The solid sample was directly placed onto an electrically conductive film after drying at 100 °C, the sample was sprayed with metal for 3 min and the morphologies of the PLY-F were observed.

### Dynamic rheology

The rheometer of Anton-Paar-MCR-102 manufactured by Austria was used to measure the high temperature and high-pressure rheological properties of polymer solution (3%) after high temperature aging 16 h (the detailed operation process is described in section “Long term aging performance”). Set the program and parameters (10 °C/10 min, 200 psi), put 30 mL polymer solution into the cup, seal the cup cover, connect the nitrogen connector, and start the program. The program is set as follows: under the condition of temperature 140 °C, 160 °C, 180 °C, the shear rate is reduced from 1000 to 5 s^−1^.

### Long term aging performance

The long-term properties of polymers were evaluated using OFITE/UNS31600 aging tanks and OFI Roller oven. Firstly, 0.35 g NaOH and 0.7 g Na_2_CO_3_ were dissolved in 250 g artificial seawater, under the condition of high-speed stirring, then adding 7.0 g PLY-F slowly and keeping high-speed stirring for 30 min.

Secondly, 205 g of potassium formate and 2.0 g of defoamer were added under high-speed stirring for 10 min. Next, pour the polymer solution (350 mL) into the OFI aging tank and keep it sealed. After filling the tank with nitrogen (400 psi) and aging in the OFI Roller oven at 200 °C for 16 h, 48 h, 72 h, respectively. The rheological properties of the system were measured by using OFI 800 rotary viscometer (the value of 600 r, 300 r, 200 r, 100 r, 6 r, 3 r) at 49 °C.

### Temperature and salt resistance boundary

The test of temperature and salt resistance of the PLY-F were similar to the steps of the long-term aging performance. Firstly, seawater and NaOH, Na_2_CO_3_ were taken into a tank to stir and dissolve at 3000 r/min. Secondly, PLY-F were added under 10,000 r/min and stirring for 30 min. After that, all kinds of salts (HCOOK/HCOONa/NaCl) and defoamer were put into the system continue stirring 30 min. the rheology performance of this system was tested by using OFI 800 rotary viscometer (the value of 600 r, 300 r, 200 r, 100 r, 6 r, 3 r) at 49 °C. Thirdly, the tested system was added to the deoxidizer and put into the aging tank which was filled with nitrogen (400 psi). Finally, the aging tank was taken out after hot rolling, then cooled and pressure released. The rheological properties of the system were tested as above at 49 °C. In addition, the amount of each component and the temperature of thermal rolling are shown in Table 2.

**Table 2:** Long term thermal stability of polymers (PLY-F).

No.	Tmperature (℃)	Density (g/cm^3^)	AV	PV	YP	YP/PV
			mPa·s	mPa·s	Pa	
1	Before	1.3	43.5	31	12.5	0.40
2	200 °C, 16 h	1.3	40.5 (93.1%)	31 (100%)	9.5 (76%)	0.30
3	200 °C, 48 h	1.3	37.5 (86.2%)	29 (93.5%)	8.5 (68%)	0.29
4	200 °C, 72 h	1.3	36 (82.7%)	28 (90.3%)	8 (64%)	0.28

## Results and discussion

### SEM photographs

**Figure 3:**
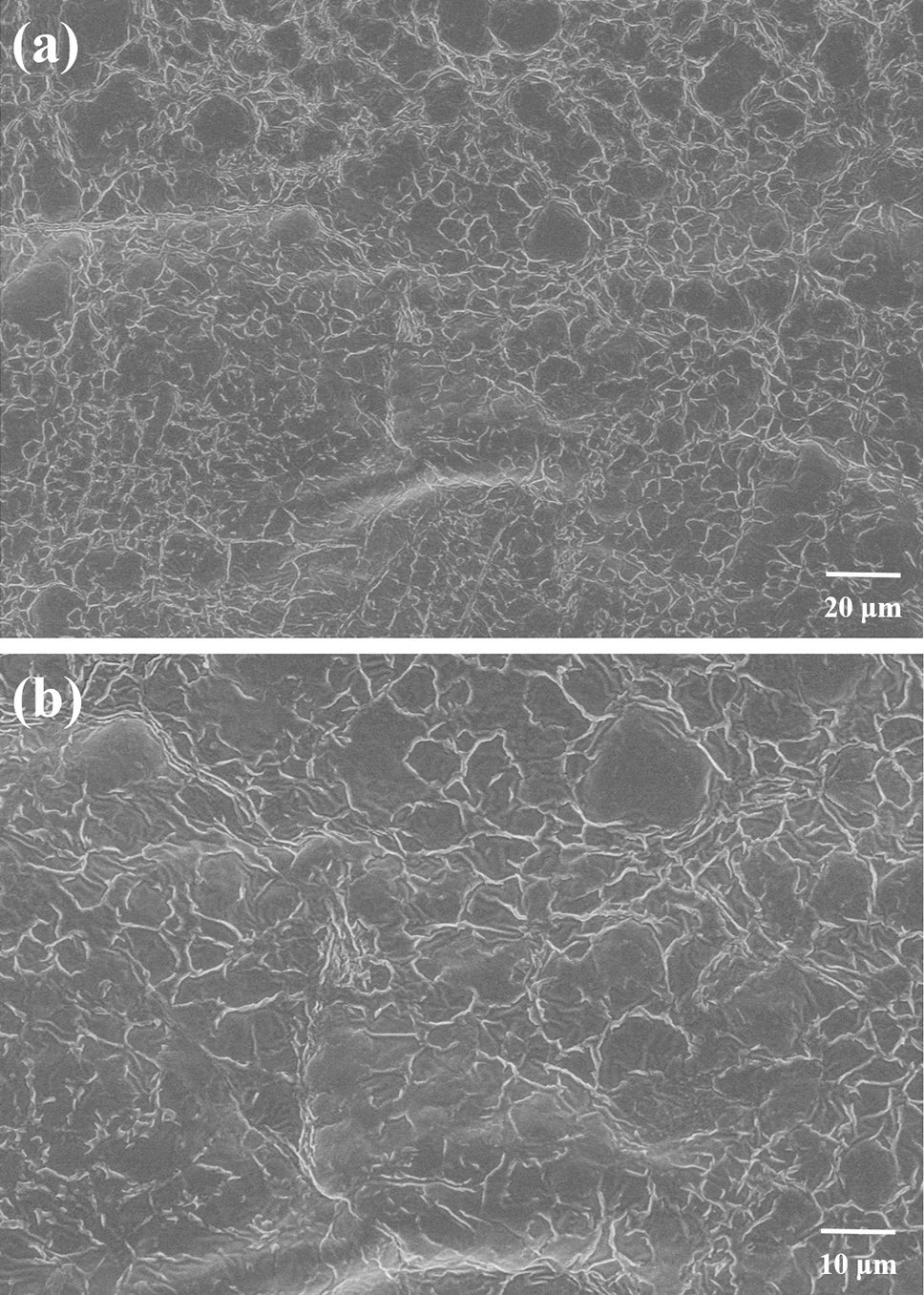
SEM images of PLY-F with different magnification (**a** 5000, **b** 10,000).

Figure 3 shows the SEM images of the polymer PLY-F. The as-synthesized polymer has a regular structure with a novel "Mesh-Lock" reinforced network cross structure as shown in Fig. 3a. The polymer shows a dense linkage, the winding structure which may make the polymer PLY-F has better stability in high temperature and high salt environment.

### FT-IR

**Figure 4:**
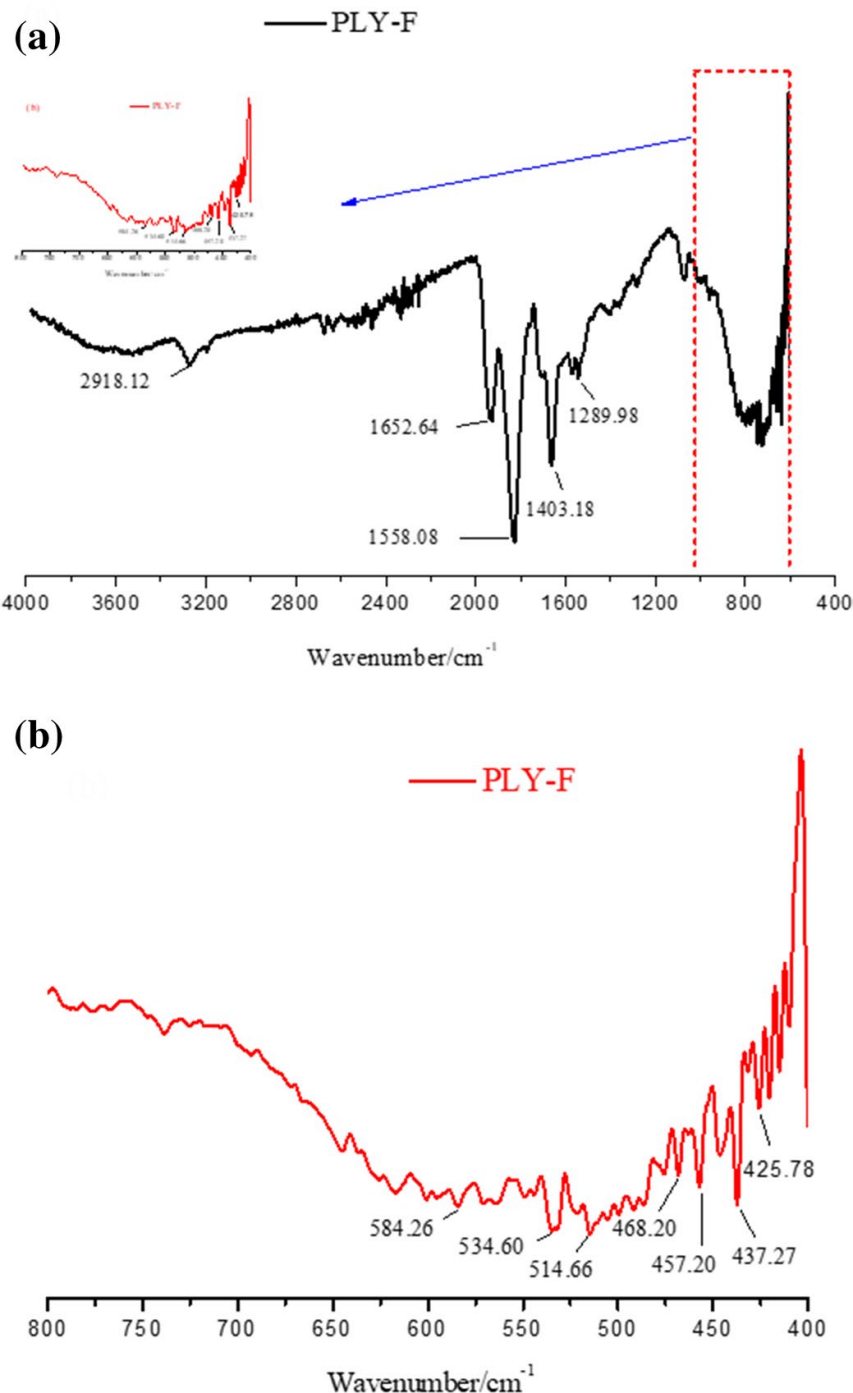
The infrared spectrum of temperature and salt-resistant polymers PLY-F.

The FTIR spectrum of polymer PLY-F was shown in Fig. 4a. A strong absorption peak of 3168 cm^−1^ was originated from the stretch vibration of the N–H bond on AMPS as shown in Fig. 3a. While the peak was observed at 2928 cm^−1^ due to the stretch vibration of –CH_3_. The peaks showed in 1652 cm^−1^, 1558 cm^−1^ and 1403 cm^−1^ were related to the stretching vibration of –C=O on NVP, the bending vibration of –NH_2_ and the stretching vibration of –C–N on AMPS, respectively. The absorption peak of stretching vibration of –C–O–C– on PAT was 1289 cm^−1^.

There was no peak at 900–1000 cm^−1^, indicating that –C=C– was all involved in the polymerization, and there was no residue in the olefin double bond. It could be concluded that the polymerization was complete and no monomers residues in polymers. Moreover, the absorption peak near 580 cm^−1^ in the fingerprint area was the bending vibration peak of multiple –CH_2_ connected by straight-chain as shown in Fig. 4b, which may be the long carbon chain of C_16_DMAAC. The results show that the temperature and salt-resistant polymer contains the characteristic functional groups of various monomers in the molecular structure.

### ^13^CNMR

**Figure 5:**
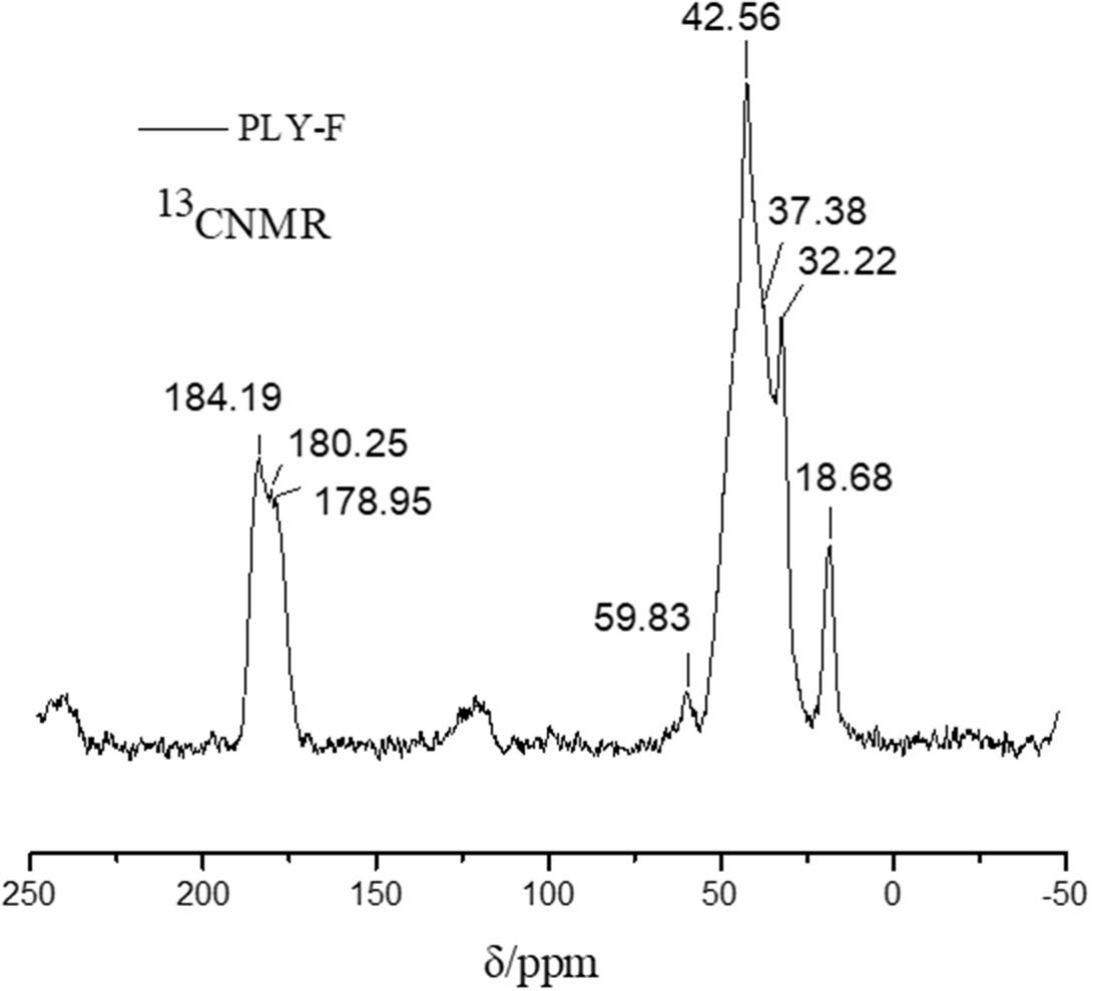
The spectrum of ^13^C NMR of temperature and salt resistant polymer PLY-F.

For further characterization of PLY-F, the ^13^CNMR of the PLY-F was performed as shown in Fig. 5. The deconvolutions of the spectrum show the characteristic signals at 32.16 and 41.86 ppm chemical shift for the resonance of the –CH_3_– and –CH_2_– carbon atom in the monomer (AM, AMPS, NVP, C_16_DMAAC, PTE). The chemical shift at 18.54 ppm is mainly due to the resonance of the –CH– carbon atom in the *N*-vinylpyrrolidone ring and indicates that NVP has reacted into the polymer. The 110–130 ppm wide chemical shifts are attributed to the CH_2_=CH– in the carbon atom in the monomer, the lower intensity of the chemical shift revealing the double bond of monomer underwent addition reaction. The strength and wide peak of the 170–190 ppm chemical shift are caused by the resonance of monomer (AM, AMPS, NVP), it revealing the three monomers have participated in the polymerization. The results of ^13^CNMR further confirmed that the polymer PLY-F has been formed and revealed the polymer was synthesis by addition polymerization through four monomers.

### Dynamic rheology

**Figure 6:**
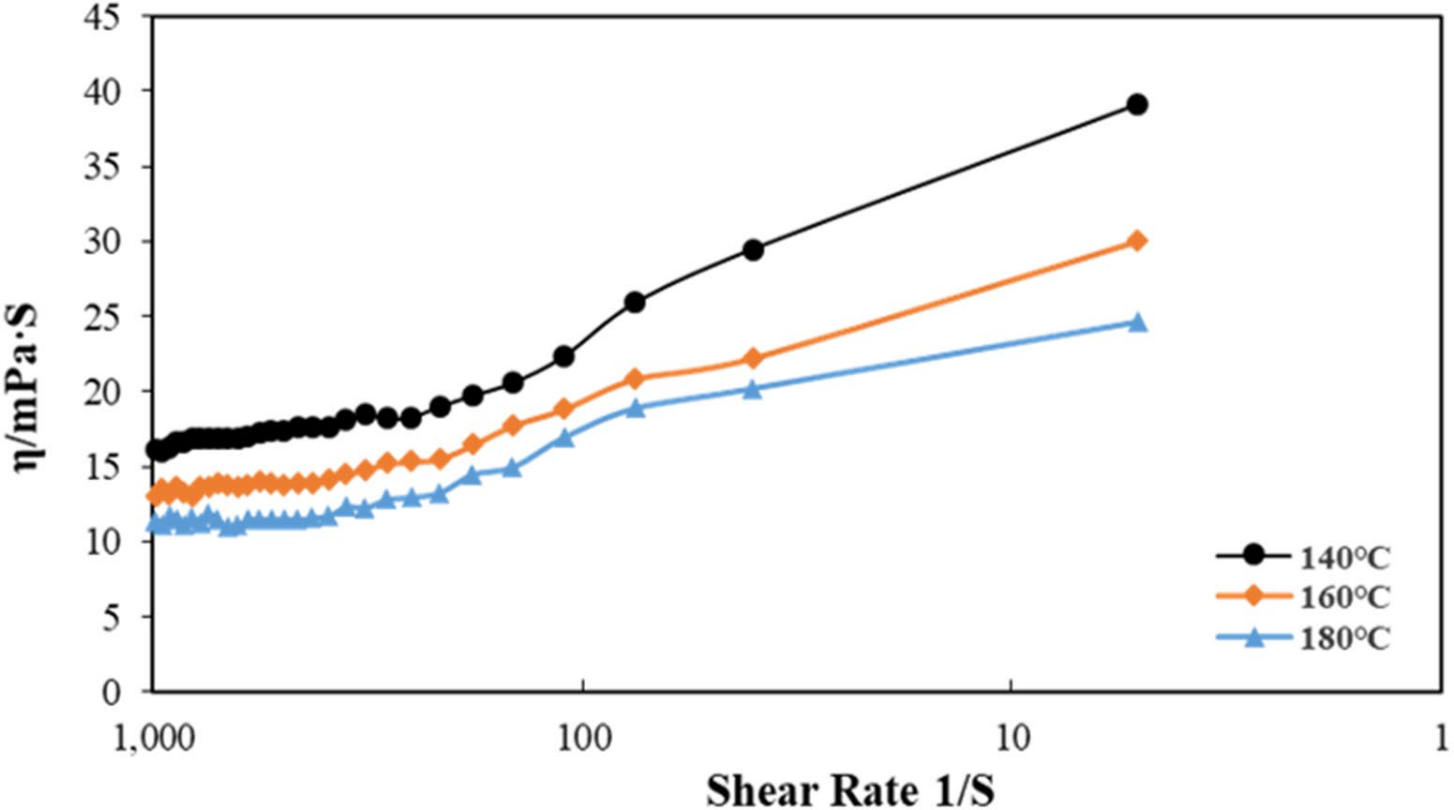
Dynamic rheology experiment of PLY-F.

In order to better grasp the rheological properties of the polymer at high temperatures, the apparent viscosity of PLY-F in a solid-free drilling fluid system was measured by using the dynamic rheometer at 140 °C, 160 °C and 180 °C, respectively. The results as shown in Fig. 6, with the decrease of shear rate (from 1000 to 5 s^−1^), the viscosity of the PLY system increases gradually, showing obvious pseudoplastic fluid characteristics.

When the temperature rises up to 180 °C, the viscosity of the system declines. However, it is more than 10 mPa·s at different shear rates which indicating that PLY-F still has good rheological properties under high temperature and high-density saline environment.

### Long term thermal stability

The purpose of adding polymer to drilling fluid is to adjust the rheology of the system, the rheology performance mainly refers to apparent viscosity (AV), plastic viscosity (PV) and dynamic shear force (YP). As shown in Table 2, the rheological properties of the drilling fluid system (250 g seawater + 7 g polymer + 205 g HCOOK) were tested after 200 °C hot rolling for 16 h, 48 h and 72 h. After heat rolling for 16 h, the AV viscosity retention rate of PLY-F solution is 93.1% and 82.7% at 72 h. Compared to AV changes, the PV retention rate is 100% for 16 h, while 90.3% for 72 h. Within 72 h, the YP value of the system converted slightly, from 9.5 to 8 Pa. YP/PV reflects the proportional relationship between structural strength and plastic viscosity of drilling fluid. The result shows that with the increase of time, the YP/PV of the system is relatively stable. As the aging time increased to 72 h, it can still reach 0.28, which indicates that the polymer PLY-F has good long-term aging performance.

### Temperature and salt resistance boundary

The rheological properties of the system were investigated after hot rolling 16 h at 200 °C and in potassium formate salt solution with a density of 1.15 g/cm^3^, 1.3 g/cm^3^, 1.4 g/cm^3^, respectively. The results were shown in Table 3.

**Table 3:** Drilling fluid system performance(using PLY-F) .

**Formula table**		
Seawater	250 mL	
PLY-F	7 g	
PF-POLYTRO (fluid loss additives)	2 g	
EZCARB (blocking agent)	10.5 g	
HCOOK (g)	205 g	
NaOH (g)	0.35 g	
Na_2_CO_3_ (g)	0.7 g	
Defoamer (g)	1 g	
Deaerator (g)	2 g	
**Performance/hot rolling 16 h (200 °C)**		
Temperature (°C)	B/200 °C	A/200 °C
Density (g/cm^3^)	1.3	
AV (mPa·s)	50	43
PV (mPa·s)	35	30
YP (Pa)	15	13
YP/PV	0.42	0.43
G″ (Pa)	4	4
G′ (Pa)	5	4
API (mL)	3.5	3.7

The polymer PLY-F (2%) in 1.4 g/cm^3^ potassium formate salt-water after 200 °C heat rolling for 16 h, the value of AV > 30 mPa·s, YP ≥ 5 Pa and YP/PV > 0.15. Obviously, the data in the table shows that the rheological properties of polymer solution become worse with the increase of salt concentration under the same hot rolling conditions (200 °C, 16 h), however, it has good performance under 1.4 g/cm^3^, the volume of 2%. As we can see, the rheological properties of the polymer increase as the salt solution density increases from 1.15 to 1.3 g/cm^3^. When the density is further increased to 1.4 g/cm^3^, the rheological properties of the polymer decrease.

The reason for that phenomenon may be the potassium formate salt water is an alkaline solution, when the potassium formate solution changes from 1.15 to 1.3 g/cm^3^, some special reactions will occur at higher concentration of formate, which makes the solution have a certain free radical capture effect, thus improving the thermal stability of the polymer^27,28^. As the formate density in the solution increases further to 1.4 g/cm^3^, the activity of water in the system decreases, which affects the solubility of the polymer, so the initial rheological properties of the polymer (before hot rolling) were affected. The rheology of the system (after hot rolling) becomes larger due to the improvement of the solubility of the polymer after thermal rolling.

As Table S1 (Supplementary materials) shows, by the condition of potassium formate solution (1.3 g/cm^3^), the rheological properties of the polymer after heat rolling 16 h under 200 °C, 210 °C, 220 °C were investigated. results show that under the condition of the 2% of the PLY-F in potassium formate solution (1.3 g/cm^3^), the maximum temperature resistance is up to 220 °C. The rheological properties of the polymer system decreased gradually with the increase of temperature, while the AV values of the system after 220 °C (1.3 g/cm^3^) thermal rolling were still above 20 mPa·s and YP/PV exceeding 0.2. Besides, the polymer still has good rheological properties in saturated solution (NaCl, HCOONa) under 210 °C. In the saturated solution of both brine, the YP of the system is higher than 20 mPa·s and YP/PV exceeds 0.4. All the above results showing the good performance of this new polymer PLY-F.

To further characterize the performance of PLY-F in drilling fluid, a water-based drilling fluid was constructed by PLY, a blocking agent and reducing agent. Moreover, the rheological value of the system was evaluated (Table 3).

As we see, The drilling fluid system prepared with PLY-F after 200 °C thermal roll has good rheological properties which reveal at AV > 40 mPa·s, YP > 10 Pa; The system still has a high value of initial gel strength and 10 min gel strength (G″/G′), besides that, the drilling fluid system also have good reduce the filter loss performance which can be seen at the lower API(3.5 mL).

The above experiments demonstrate that PLY-F is suitable as a high temperature and salt adhesive for water-based solid-free drilling fluid.

### Mechanism of temperature and salt resistance

**Figure 7:**
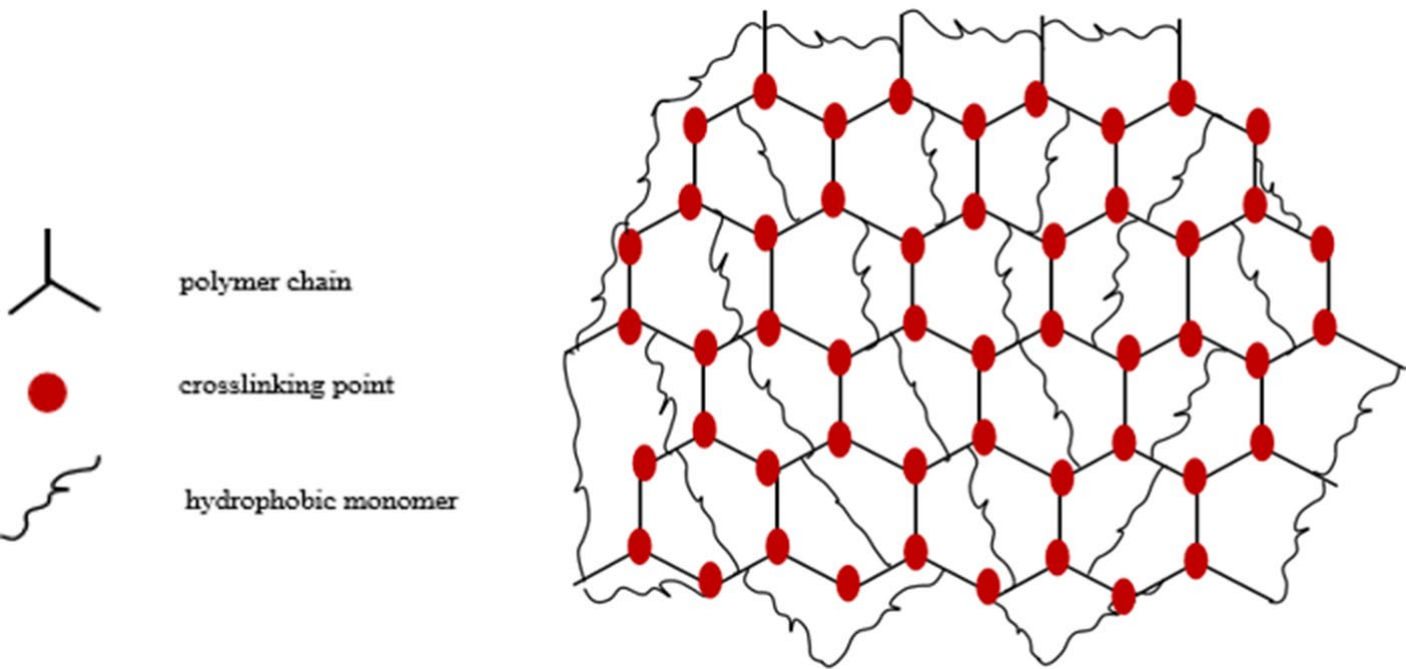
Cross structure diagram of polymer PLY-F reinforcement network.

It is well known that the polymer molecular chains will curl up and damage at high temperatures and high salt environments. In short, the temperature and salt resistance mechanism of PLY-F is called “Mesh-Lock”. “Mesh” means that when a copolymer AM, AA, NVP are in an aqueous solution for free radical polymerization chain growth, the crosslinking agent (PTE) synchronizes with the monomer through three double bonds in its molecular structure. The effect of crosslinking makes the polymer molecular chains winding together to form a certain degree of spatial "network" were filled with a large number of hydrophobic chains. Meanwhile, the distance between polymer molecules is narrowed due to the crosslinking agent, so that the non-covalent bond force between hydrophobic monomers on a molecular chain is enhanced as shown in Fig. 7. The mesh of polymer molecules is further tightened such as ("Lock"), thus improving the stability of polymer chains as a whole.

## Conclusions

In this paper, the preparation and characterization of a temperature and salt-resistant polymer for solid-free water-based drilling fluid were introduced. In summary, the polymer PLY-F was obtained by these substances as AM, AMPS, AA, NVP, PTE and C_16_DMAAC with free-radical polymerization in an aqueous solution. Especially, the "Mesh-Lock" structure formed by intermolecular crosslinking and hydrophobic interaction makes the molecular configuration relatively stable in high temperature and high salt environments. This polymer PLY-F remained stable in 1.4 g/cm^3^ potassium formate salt-water for 200 °C and the performance of temperature resistance to 220 °C at 1.3 g/cm^3^ potassium formate solution. In addition, the PLY-F still has good rheological properties in saturated solution (NaCl or HCOONa) under 210 °C. Obviously, the PLY-F is a novel polymer that can be used as a viscosifying agent in solid-free water-based drilling fluid under high temperature and high-density surroundings.

## Supplementary Information

Supplementary Table S1.
