# Supplementary material for: Fabrication of a state of the art mesh lock polymer for water based solid free drilling fluid
Source: Sci Rep. 2021 Sep 22;11:18870. doi: 10.1038/s41598-021-98379-w (PMC8458390; doi:10.1038/s41598-021-98379-w)
Supplement: Supplementary file 1 — Supplementary Table S1. [file 41598_2021_98379_MOESM1_ESM.docx]

***Supplementary materials***

Table S1 Temperature and salt resistance performance of PLY-F

| Formulation | | | | | | | | | | | | |
| --- | --- | --- | --- | --- | --- | --- | --- | --- | --- | --- | --- | --- |
| Seawater/ g | 301.9 | | 250 | | 210 | | 250 | 250 | 290 | | 260 | |
| PLY-F/ g | 7.00 | | 7.00 | | 7.00 | | 7.00 | 7.00 | 7.00 | | 7.00 | |
| HCOOK/ g | 100.6 | | 205 | | 279 | | 205 | 205 | NaCl (102 g) | | HCOONa (195 g) | |
| NaOH/ g | 0.35 | | 0.35 | | 0.35 | | 0.35 | 0.35 | 0.35 | | 0.35 | |
| Na_2_CO_3_/ g | 0.7 | | 0.7 | | 0.7 | | 0.7 | 0.7 | 0.7 | | 0.7 | |
| Defoamer/ g | 2.0 | | 2.0 | | 2.0 | | 2.0 | 2.0 | 2.0 | | 2.0 | |
| Deaerator/ g | 1.0 | | 1.0 | | 1.0 | | 1.0 | 1.0 | 1.0 | | 1.0 | |
| Density/ g·cm^-1^ | 1.15 | | 1.3 | | 1.4 | | 1.3 | 1.3 | Saturated | | Saturated | |
| Performance/Hot rolling 16 h | | | | | | | | | Performance/ Hot rolling 16 h  (Saturated saline) | | | |
| Temperature/℃ | B/200℃ | A/200℃ | B/200℃ | A/200℃ | B/200℃ | A/200℃ | A/210℃ | A/220℃ | B/210℃ | A/210℃ | B/210℃ | A/210℃ |
|  | 49℃ | 49℃ | 49℃ | 49℃ | 49℃ | 49℃ | 49℃ | 49℃ | 49℃ | 49℃ | 49℃ | 49℃ |
| Ф600 | 154.0 | 104.0 | 165.0 | 125.0 | 43.0 | 61.0 | 81.0 | 53.0 | 121.0 | 120.0 | 164.0 | 166.0 |
| Ф300 | 109.0 | 69.0 | 115.0 | 80.0 | 24.0 | 35.0 | 50.0 | 31.0 | 91.0 | 80.0 | 108.0 | 107.0 |
| Ф200 | 88.5 | 53.0 | 91.0 | 61.0 | 16.0 | 25.0 | 37.0 | 22.0 | 73.0 | 62.0 | 81.0 | 82.0 |
| Ф100 | 61.5 | 33.0 | 61.0 | 38.0 | 8.5 | 14.0 | 21.0 | 12.0 | 48.0 | 40.0 | 50.0 | 51.0 |
| Ф6 | 10.0 | 3.0 | 9.0 | 4.0 | 0.5 | 1.0 | 1.5 | 1.0 | 8.0 | 4.0 | 5.0 | 6.0 |
| Ф3 | 7.0 | 2.0 | 6.0 | 2.0 | 0.0 | 0.0 | 1.0 | 0.5 | 5.0 | 2.5 | 3.0 | 3.0 |
| AV/(mPa·s) | 77 | 52 | 83 | 63 | 22 | 31 | 41 | 27 | 61 | 60 | 82 | 83 |
| PV/(mPa·s) | 45 | 35 | 50 | 45 | 19 | 26 | 31 | 22 | 30 | 40 | 56 | 59 |
| YP/Pa | 32 | 17 | 33 | 18 | 3 | 5 | 10 | 5 | 31 | 20 | 26 | 24 |
| YP/PV | 0.71 | 0.49 | 0.65 | 0.39 | 0.13 | 0.17 | 0.31 | 0.21 | 1.04 | 0.50 | 0.46 | 0.41 |
| B:before hot rolling, A: after hot rolling | | | | | | | | | | | | |
